# Sublethal concentrations of 17-AAG suppress homologous recombination DNA repair and enhance sensitivity to carboplatin and olaparib in HR proficient ovarian cancer cells

**DOI:** 10.18632/oncotarget.1929

**Published:** 2014-04-30

**Authors:** Young Eun Choi, Chiara Battelli, Jacqueline Watson, Joyce Liu, Jennifer Curtis, Alexander N. Morse, Ursula A. Matulonis, Dipanjan Chowdhury, Panagiotis A. Konstantinopoulos

**Affiliations:** ^1^ Department of Radiation Oncology, Dana Farber Cancer Institute, Harvard Medical School; ^2^ Maine Center for Cancer Medicine, Scarborough, Maine; ^3^ Department of Medical Oncology, Medical Gynecologic Oncology Program, Dana Farber Cancer Institute, Harvard Medical School; ^4^ New York University Global Institute of Public Health

**Keywords:** Epithelial ovarian cancer, platinum, PARP inhibitors, Heat Shock Protein 90 inhibitors, homologous recombination

## Abstract

The promise of PARP-inhibitors(PARPis) in the management of epithelial ovarian cancer(EOC) is tempered by the fact that approximately 50% of patients with homologous recombination (HR)-proficient tumors do not respond well to these agents. Combination of PARPis with agents that inhibit HR may represent an effective strategy to enhance their activity in HR-proficient tumors. Using a bioinformatics approach, we identified that heat shock protein 90 inhibitors(HSP90i) may suppress HR and thus revert HR-proficient to HR-deficient tumors. Analysis of publicly available gene expression data showed that exposure of HR-proficient breast cancer cell lines to HSP90i 17-AAG(17-allylamino-17-demethoxygeldanamycin) downregulated HR, ATM and Fanconi Anemia pathways. In HR-proficient EOC cells, 17-AAG suppressed HR as assessed using the RAD51 foci formation assay and this was further confirmed using the Direct Repeat-GFP reporter assay. Furthermore, 17-AAG downregulated BRCA1 and/or RAD51 protein levels, and induced significantly more γH2AX activation in combination with olaparib compared to olaparib alone. Finally, sublethal concentrations of 17-AAG sensitized HR-proficient EOC lines to olaparib and carboplatin but did not affect sensitivity of the HR-deficient OVCAR8 line arguing that the 17-AAG mediated sensitization is dependent on suppression of HR. These results provide a preclinical rationale for using a combination of olaparib/17-AAG in HR-proficient EOC.

## INTRODUCTION

Epithelial ovarian cancer (EOC) is characterized by frequent genetic and epigenetic alterations in gene members of the homologous recombination (HR) DNA repair pathway. In particular, approximately 50% of high grade serous cancers harbor molecular alterations in the HR pathway which include germline and somatic BRCA1/2 mutations in 15% and 6-7% of them respectively [1, 2]. HR deficient tumors are highly sensitive to platinum analogues and other cytotoxic drugs that induce double strand DNA breaks which are normally repaired by HR. These tumors are also highly sensitive to poly-ADP ribose polymerase inhibitors (PARPis), a novel class of anticancer agents, which exhibit synthetic lethality in tumors with defective HR pathway [3-5]. PARPis have shown striking activity in HR-deficient EOC tumors both in the presence [6-8] and in the absence of BRCA1 or BRCA2 mutations [9, 10]. Of these agents, olaparib has been the most widely studied PARPi and is currently in the most advanced stage of clinical development [6, 7, 9-11].

The promise of PARP inhibitors in the management of EOC is tempered by the fact that HR-proficient EOCs do not respond well to these agents, suggesting that the remaining approximately 50% of EOC patients (i.e. those without HR alterations) do not benefit from this novel class of drugs. Combination of PARPis with agents that inhibit HR may represent an effective strategy to enhance activity of PARPis in HR proficient tumors and thus potentially expand use of these agents beyond patients with HR deficient EOCs.

In order to identify candidate agents that may directly or indirectly inhibit HR, we used the Connectivity Map, a reference collection of gene expression signatures that have been generated by exposing cultured human cell lines to a variety of small molecule drugs[12, 13]. By matching gene expression signatures of disease states or physiological processes with gene expression signatures of small molecule drugs, the Connectivity Map has successfully unraveled novel functional associations between small molecules sharing a mechanism of action, chemicals and physiological processes, and diseases and drugs. Here, we report that, using the Connectivity Map we identified heat shock protein 90 inhibitors (HSP90i) as candidate drugs that suppress HR, and then confirmed experimentally that an HSP90i 17-AAG[14, 15] suppresses HR pathway, and enhances sensitivity to platinum and PARPis in HR proficient ovarian cancer cells.

## RESULTS

### Connectivity Map identifies HSP90is as candidate compounds that suppress HR

**FIGURE 1:**
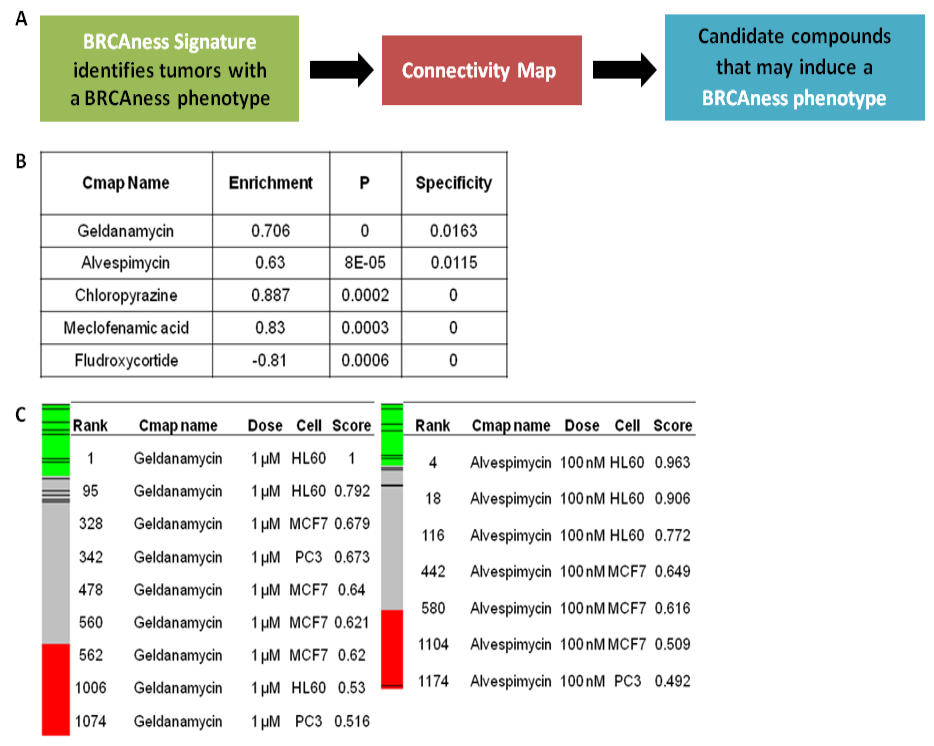
Application of BRCAness signature into Connectivity Map identifies HSP90 inhibitors as candidate compounds that may suppress HR **(A)** Schematic of the bioinformatics approach used to identify candidate compounds that may suppress of HR. **(B)** Top ranked compounds, enrichment, permutation p and specificity values as determined by query of the top performing genes of the BRCAness signature. **(C)** Connectivity mapping of geldanamycin and alvespimycin. The barview is constructed from 6,100 horizontal lines, each representing an individual treatment instance, ordered by their corresponding connectivity scores with geldanamycin (left) and alvespimycin (right). All geldanamycin and alvespimycin instances are colored in black bars. Colors applied to the remaining instances (i.e. gene expression profiles of the cells obtained with other than geldanamycin and alvespimycin) reflect the sign of their scores (green,positive; gray, null; red, negative). The rank, concentration, cell line and connectivity score for geldanamycin and alvespimycin are also shown.

We queried a previously developed gene expression signature of BRCAness[16] to the Connectivity Map in order to identify matching gene expression signatures of small molecule drugs (Figure 1A). Because of the potential association of the BRCAness gene expression signature with defective HR, we hypothesized that drugs identified via the Connectivity Map whose gene expression signatures are similar to the BRCAness signature may be functionally associated with induction of defective HR and thus reversion of HR proficient to HR deficient tumors.

Application of the top performing genes of the BRCAness signature to the Connectivity Map identified a number of interesting compounds (Figure 1B) with high connectivity scores across several cell lines included in the Connectivity Map (Figure 1C). Of note, the two highest performing compounds, which were consistently identified using various cut-offs for the top performing genes of BRCAness signature, were geldanamycin and alvespimycin, both HSP90 inhibitors. This finding raised the possibility of a functional relationship between HSP90 inhibition and induction of deficient HR. In this regard, as predicted by the Connectivity Map, we hypothesized that these agents might enhance PARPi and platinum sensitivity by suppressing HR (either directly or indirectly) thereby suggesting that HSP90is may have an off-target class effect involving the HR pathway.

### 17-AAG downregulates HR pathway and gene expression in HR proficient but not HR deficient cells

Given that the Connectivity Map identified HSP90is as candidate agents that suppress HR, we evaluated whether the transcriptional response to HSP90is affects HR pathway and HR pathway genes in cancer cell lines. We queried GEO for publicly available gene expression data of response to HSP90is and identified one microarray dataset assessing the transcriptional response of breast cancer cell lines to HSP90i 17-AAG (17-allylamino-17-demethoxygeldanamycin). 17-AAG is an HSP90 inhibitor that has been evaluated in clinical trials in various malignancies, and is currently in the most advanced stage of clinical development of all HSP90is. Furthermore, unlike geldanamycin, it is not hepatotoxic and is therefore appropriate for clinical use.

This microarray dataset included gene expression data of the HR proficient (Hs578T, MCF-7, MDA-MB-157, T47D and MDA-MB-231) and HR deficient (MBA-MD-436, HCC-1937 and UACC3199) breast cancer cell lines exposed to 17-AAG or control. We assessed 17-AAG induced changes in the expression levels of genes of the HR pathway both at an individual gene level and at a global pathway level.

Strikingly, in the HR proficient breast cancer cell lines, pathway analysis revealed that exposure to 17-AAG statistically significantly downregulated HR (p<0.005), ATM (p=0.015) signaling and Fanconi Anemia (p<0.005) pathways, which are involved in repair of double strand breaks and interstrand crosslinks (Table 1). Furthermore, the expression levels of several genes of these pathways were decreased upon exposure to 17-AAG (Table 1). Conversely, in the HR deficient (MBA-MD-436, HCC-1937 and UACC3199) breast cancer cell lines, 17-AAG did not significantly affect any of these pathways (p value non significant for all 3 aforementioned pathways).

**TABLE 1:** 17-AAG down-regulates HR pathway genes in HR competent breast and ovarian cancer cells

HR competent Breast Lines (Hs578T, MCF-7, MDA-MB-157, MDA-MB-231)	Pathways Up in control vs 17-AAG treated cells	Pathway P	Genes	% Up in control vs 17-AAG treated cells
	KEGG HOMOLOGOUS RECOMBINATION	<0.005	FAA100	69
	REACTOME FANCONI ANEMIA PATHWAY	0.005	FANCL	68
	BIOCARTA ATM SIGNALING PATHWAY	0.015	RAD51C	58
			RPA3	55
			BRCA1	50
			BLM	50
			FANCA	37
			FANCC	33
			ATR	32
			FANCG	31
			NBN	28
			RAD54L	27
			RAD51	22

### Sublethal concentrations of 17-AAG suppress HR in HR proficient ovarian cancer cell lines

**FIGURE 2:**
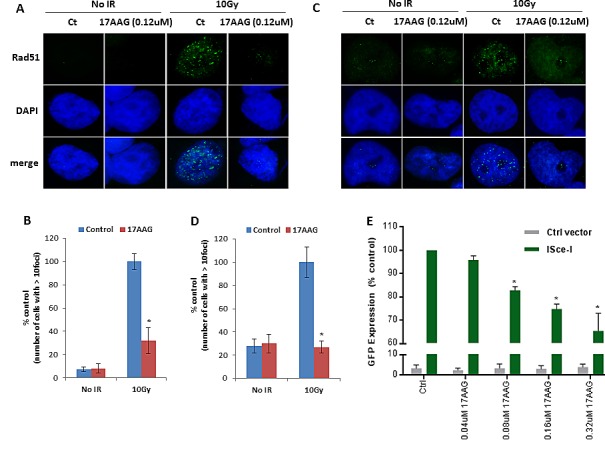
17-AAG downregulates HR-mediated DSB repair **(A-D)** Analysis of HR-mediated repair by RAD51 focus formation. 36M2 cells (A and B) or SKOV3 cells (C and D) were treated with 17-AAG or vehicle control for 24 hrs, stained for RAD51 (green) and 4′,6-diamidino-2-phenylindole (DAPI) (blue) 6 h after exposure to IR. The images were captured by fluorescence microscopy and RAD51 focus-positive cells (with > 10 foci) were quantified by comparing 100 cells. Mean ± SD of 3 independent experiments is graphically represented in Figure 2C and 2E. * indicates p< 0.05. **(E)** Measurement of HR-mediated repair of an I-SceI induced site specific DSB. Cells carrying a single copy of the recombination substrate (DR-GFP) were treated with indicated concentrations of 17-AAG or vehicle control for 24 hrs before transfection with I-SceI or control vector. GFP positive cells were analyzed 48 h later by flow cytometry (FACS). Mean ± SD of 3 independent experiments is graphically represented. * indicates p< 0.05.

RAD51 is involved in HR, and formation of nuclear RAD51 foci after IR has been used as a surrogate of effective HR DNA repair. We evaluated the functional impact of 17-AAG on HR in HR proficient ovarian cancer cell lines using the RAD51 foci formation after ionizing radiation (IR) assay. As shown in Figure 2A-2D, sublethal concentrations of 17-AAG (as determined by 17-AAG dose response curves that were obtained for each ovarian cancer cell line, Supplementary Figure 1) had a significant impact on RAD51 foci after IR causing approximately 70% reduction in foci formation in 36M2 and SKOV3 cells.

To further confirm the results of the RAD51 foci formation assay we used the Direct Repeat-GFP (DR-GFP) reporter system previously developed to assay HR in mammalian cells [17, 18]. DR-GFP is an integrated fluorescence-based reporter that allows for the efficient quantification of HR at a single, targeted DSB by flow cytometry or immunofluorescence microscopy. As shown in Figure 2E, 17-AAG diminished HR-mediated DSB repair in a dose dependent fashion.

### 17-AAG downregulates RAD51 and/or BRCA1 protein levels in ovarian cancer cells

**FIGURE 3:**
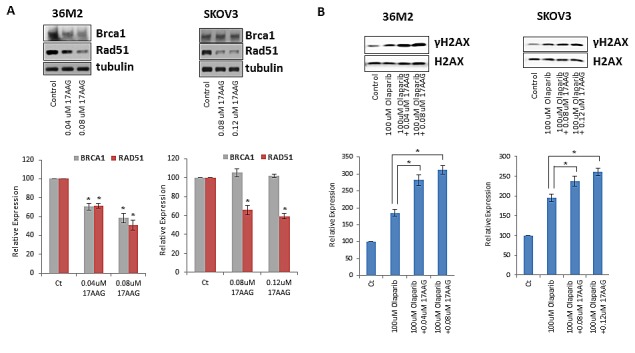
17-AAG downregulates the genes involved in HR pathway **(A)** Expression of HR genes is impacted by 17-AAG. Indicated cells were treated with 17-AAG or vehicle control for 24 hrs and washed off before subjected to immunoblotting in another 24 hrs. Cell lysates were analyzed by immunoblot for BRCA1 or RAD51. Images were quantified by ImageJ software and the mean ± SD of 3 independent experiments is graphically shown in the lower panel, * indicates p< 0.05. **(B)** γ-H2AX accumulation after treatment with olaparib ± 17-AAG. Indicated cells were treated with olaparib ± 17-AAG for 24 hrs before evaluation of γ-H2AX by immunoblotting. Total H2AX was served as loading control for these experiments. * indicates p< 0.05.

We evaluated whether 17-AAG downregulates HR pathway genes in HR proficient ovarian cancer cell lines as predicted by the microarray data in the breast cancer cell lines (Table 1). For this purpose, we performed Western blot of two representative HR pathway genes, RAD51 and BRCA1 that were found to be downregulated upon exposure to 17-AAG in HR proficient cells (Table 1). As shown in Figure 3A, 17-AAG downregulated protein levels of BRCA1 and RAD51 in 36M2 and RAD51 alone in SKOV3 ovarian cancer cell lines.

### Sublethal concentrations of 17-AAG combined with olaparib induce γH2AX activation more than olaparib alone in ovarian cancer cells

Having demonstrated that 17-AAG functionally inhibits HR, we determined whether 17-AAG could sensitize ovarian cancer cells to the cytotoxic effects of PARPi olaparib. We first assessed γH2Ax activation which is a surrogate of DNA damage and a marker of cytotoxicity of ovarian cancer cells. Specifically, we examined whether γH2AX expression was enhanced by the combination of 17-AAG and olaparib compared to olaparib alone. As shown in Figure 3B, we confirmed enhanced γH2AX expression in the HR proficient ovarian cancer cells 36M2 and SKOV3 treated with sublethal concentrations of 17-AAG in combination with olaparib compared to olaparib alone.

### 17-AAG sensitizes HR proficient ovarian cancer cell lines to olaparib and carboplatin *in vitro*

**FIGURE 4:**
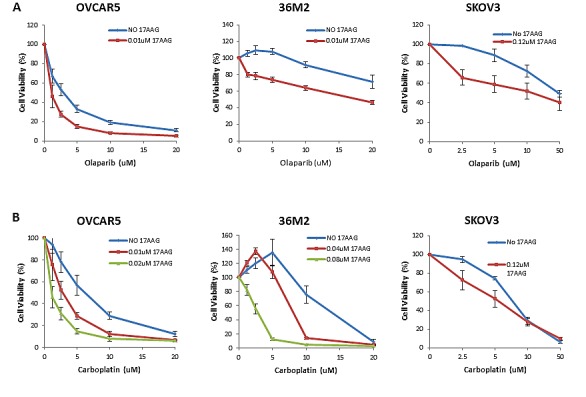
17-AAG sensitizes HR-proficient cells to olaparib and carboplatin **(A and B)** Luminescence-based viability assay in HR-proficient cells with olaparib or carboplatin. Cells were plated onto a 96-well plate at 1000 cells/well density and treated with indicated concentrations of PARP inhibitor, olaparib (A) or platinum drug, carboplatin (B) on the following day. Viability was tested by using CellTiter Glo (Promega) in 5 days. Curves were generated from 3 independent experiments.

In order to assess whether 17-AAG induces sensitivity to olaparib we used only sublethal doses of 17-AAG as determined in the dose responses curves in each cell line (Supplementary Figure 1). As shown in Figure 4A, sublethal concentrations of 17-AAG were associated with increased sensitivity of HR proficient cell lines SKOV3, OVCAR5 and 36M2 cells to olaparib. Furthermore, exposure of these HR proficient ovarian cancer cell lines to sublethal concentrations of 17-AAG also enhanced sensitivity to carboplatin (Figure 4B). Finally, viability assays with a combination of three drugs, olaparib, carboplatin, and 17-AAG, in HR-proficient ovarian cancer cell lines (36M2 and OVCAR5) showed that 17-AAG also enhanced sensitivity to the combination of olaparib and carboplatin. Of note, for both OVCAR5 and 36M2 cells, we were able to detect significant killing with very low olaparib and carboplatin doses, i.e. as low as 2.5uM (Supplementary Figure 2).

### 17-AAG does not sensitize HR deficient OVCAR8 cell line to olaparib and carboplatin *in vitro*

**FIGURE 5:**
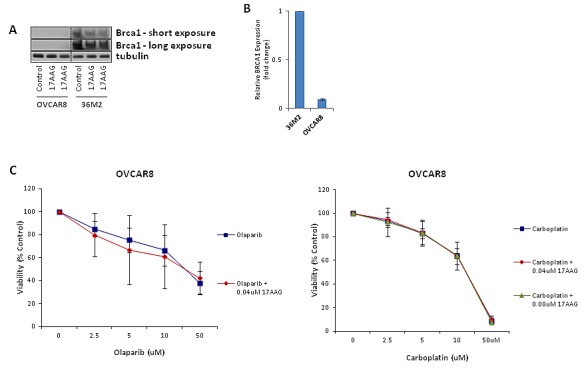
17-AAG does not sensitize HR-deficient cells to olaparib or carboplatin **(A and B)** Validation of undetectable levels of BRCA1 expression in OVCAR8 cells. OVCAR8 cells were analyzed for BRCA1 expression by immunoblotting (A) and qRT-PCR (B) compared to BRCA1-proficient 36M2 cells.**(C)** Viability assay in HR-deficient cells with PARP inhibitor or platinum drug. Viability assay was done in the same way as in Figure 4.

We previously showed that 17-AAG suppresses HR and enhances sensitivity of HR proficient cell lines to olaparib and carboplatin. In order to evaluate the effect of 17-AAG in HR deficient ovarian cancer cells, we used the OVCAR8 cell line which harbors almost undetectable levels of BRCA1 protein (Figure 5A) and very low levels of BRCA1 transcript (Figure 5B) compared to 36M2 ovarian cancer line. As shown in Figure 5C, sublethal concentrations of 17-AAG did not sensitize OVCAR8 cells to olaparib or carboplatin.

## DISCUSSION

Although platinum analogues and PARPis exhibit striking activity in HR deficient EOCs, HR proficient tumors do not respond well to these agents. Furthermore, acquired resistance to PARPis in HR deficient EOCs frequently occurs via restoration of HR; for example, BRCA1/2-mutated EOCs become HR proficient via secondary BRCA1/2 mutations that restore BRCA1/2 function and lead to PARPi and platinum resistance [19-23]. Therefore, combination of PARPis with agents that inhibit HR may represent an effective strategy to enhance activity of PARPis in EOCs that are HR proficient either at baseline or at the time of development of platinum or PARPi resistance. In this regard, CDK1 inhibitors and PI3K inhibitors, which inhibit HR, have been shown to sensitize HR proficient cells to PARP inhibitors [24, 25].

In this study, we used a unique bioinformatics approach to identify novel candidate agents that have the potential to suppress HR and thus revert HR proficient to HR deficient tumors. Application of our previously developed, publicly available, BRCAness signature to the Connectivity Map consistently identified HSP90is as the highest performing compounds, thus raising the possibility of a functional relationship between HSP90 inhibition and induction of HR deficiency. To test this hypothesis, we first accessed publicly available microarray data of the transcriptional response of cancer cell lines to the HSP90i 17-AAG, and found that exposure to 17-AAG statistically significantly downregulated HR, ATM and Fanconi Anemia signaling pathways both at the level of the pathway and at the level of individual genes. Interestingly, this effect was only evident in the HR-proficient but not in HR-deficient breast cancer cells. We then confirmed experimentally that 17-AAG functionally suppressed HR pathway using both the DR-GFP reporter and RAD51 foci formation assays after IR. Of note, even sublethal concentrations of 17-AAG had a significant functional impact on HR causing approximately 70% reduction in RAD51 foci formation in HR proficient ovarian cancer cell lines. Importantly, 17-AAG decreased BRCA1 and RAD51 protein levels in HR proficient EOC cells.

Given that 17-AAG suppressed HR, we assessed whether 17-AAG would sensitize EOC cells to platinum and PARPis. Indeed, we showed that the combination of sublethal concentrations of 17-AAG with olaparib induced greater DNA damage (assessed by γH2AX expression) compared to olaparib alone. Furthermore, sublethal concentrations of 17-AAG enhanced cytotoxicity of HR proficient EOC cells to carboplatin and olaparib. Of note, 17-AAG also enhanced sensitivity to the combination of olaparib and carboplatin inducing significant killing even with very low olaparib and carboplatin doses (Supplementary Figure 2). Conversely, 17-AAG did not enhance cytotoxicity of the HR deficient OVCAR8 cell line to carboplatin and olaparib. This finding strongly suggests that the 17-AAG induced sensitization to olaparib and carboplatin is related to suppression of HR and not due to another (HR-independent) mechanism. Although our data suggest that 17-AAG suppresses HR and sensitizes to olaparib and carboplatin *in vitro*, we would like to recognize that 17-AAG-induced sensitization to platinum and PARP-inhibitors need to be further confirmed using in vivo models.

It is important to underscore that the 17-AAG doses of 10-100nM that were used in our experiments were sublethal in the ovarian cancer cell lines employed in our study although they are reportedly fully cytostatic in other settings (Ref. 26-28). One reason may be because HSP90-inhibitors induce HSP70 (a known pharmacodynamic marker of 17-AAG therapy) which bears anti-apoptotic effects [26-28]. Therefore, 17-AAG may exert dual effects on cell death that is cell-context-dependent and dependent on drug combinations [26].

In conclusion, sublethal concentrations of the HSP90i 17-AAG suppress HR and enhance sensitivity of HR proficient ovarian cancer cells to platinum and PARPis. These results provide a preclinical rationale for using a combination of 17-AAG and olaparib and/or carboplatin in EOCs that are HR proficient either at baseline or at the time of development of platinum or PARPi resistance.

## MATERIALS AND METHODS

### Connectivity Map Analysis

We used our previously developed, publicly available, gene expression signature of BRCAness[16] which assigned tumors as having a BRCAness phenotype (BRCAlike-BL) versus not (non-BRCAlike-NBL). In order to identify matching gene expression signatures of small molecule drugs, we queried the Connectivity Map using the top performing genes of the BRCAness signature, i.e. those that were most upregulated and downregulated in BL versus NBL tumors using various cutoffs for differential expression of these genes (i.e. 2 fold, 1.66 fold and 1.5 fold). Small molecule drugs that were consistently identified via the Connectivity Map across all cut-offs were then selected for experimental validation as candidate suppressors of the HR pathway. Details of the Connectivity Map dataset and analytics are provided elsewhere [12, 13].

### Microarray data analysis

We accessed Agilent-014850 Whole Human Genome Microarray expression data of 17-AAG treated breast cancer cell lines[29] that were publicly available in GEO (Gene Expression Omnibus, accession #23209). Raw data were imported and analyzed using BRB-ArrayTools Version: 4.2.0 - Beta_2 (Biometrics Research Branch, National Cancer Institute). In order to assess whether the gene expression profiles of control and 17-AAG treated breast cancer cell lines were enriched for specific pathways or functional groups of genes, we performed gene set analysis (GSA) as described by Efron and Tibshirani[30]. GSA is an evolution of the previously reported Gene Set Enrichment Analysis (GSEA) and was performed using the Gene Set Analysis Tool of the Biometric Research Branch (BRB) Array Tools software. Gene Ontology assignments, Biocarta and KEGG pathways, and annotated gene sets from The Broad Institute Molecular Signatures Database (MSigDB) were analyzed. The Efron-Tibshirani “maxmean” test was applied to identify gene sets at a GSA P<0.05 significance level.

### Cell Viability assay

Cells were plated at 1000 cells per well on a 96-well plate in sextuplicate and treated with PARP inhibitor and/or carboplatin with or without 17 AAG at indicated concentrations on the next day. After 5 days, cell viability was quantified by Celltiter Glo.

### Homologous Recombination Reporter Assay

HR assay was performed as previously reported [17, 18, 31]. Briefly, 0.1 × 10^6^ U2OS cells carrying DR-GFP reporter were plated on a 12-well plate overnight, treated with 17 AAG at indicated concentrations for 24 hr, and transfected with 500 ng of I-SceI expression plasmid using Lipofectamine 2000. After 48 h, GFP-positive cells were assayed by FACScan.

### Immunofluorescence

Cells plated on glass slides were fixed for 10 min with 4% (v/v) paraformaldehyde and permeabilized for 10 min with 1% (v/v) Triton X-100 in PBS. Cells were rinsed with PBS and incubated with RAD51 (Santa Cruz) primary antibody diluted in PBS with 5% goat serum for 2 hrs at room temperature (RT). Cells were washed, incubated with secondary antibody (Alexa Fluor 488 goat anti-rabbit IgG, Invitrogen) diluted in PBS with 5% goat serum for 1 hr at RT in the dark, and washed before being mounted using Dapi Fuoromount-G (SouthernBiotech). Slides were visualized by Zeiss Axioplan microscope and RAD51 focus-positive cells (with > 10 foci) were quantified by comparing 100 cells.

### Immunoblots

The immunoblots were done as described previously [32, 33] with BRCA1 (Calbiochem #OP92), RAD51 (Santa Cruz #sc-8349), H2AX (Cell Signaling #2595S), γ-H2AX (Cell Signaling #9718S) and α-tubulin (Sigma #T5168) antibodies.

### Funding

This work was supported by the Ovarian Cancer Research Fund Program Project Grant to Dr Matulonis and Dr Konstantinopoulos.

## SUPPLEMENTARY FIGURES


