# Supplementary material for: Sublethal concentrations of 17-AAG suppress homologous recombination DNA repair and enhance sensitivity to carboplatin and olaparib in HR proficient ovarian cancer cells
Source: Oncotarget. 2014 Apr 30;5(9):2678–87. doi: 10.18632/oncotarget.1929 (PMC4058036; doi:10.18632/oncotarget.1929)
Supplement: Supplementary file 1 [file oncotarget-05-2678-s001.pdf]

Sublethal concentrations of 17-AAG suppress homologous recombination DNA repair and enhance sensitivity to carboplatin and olaparib in HR proficient ovarian cancer cells

Supplementary Information

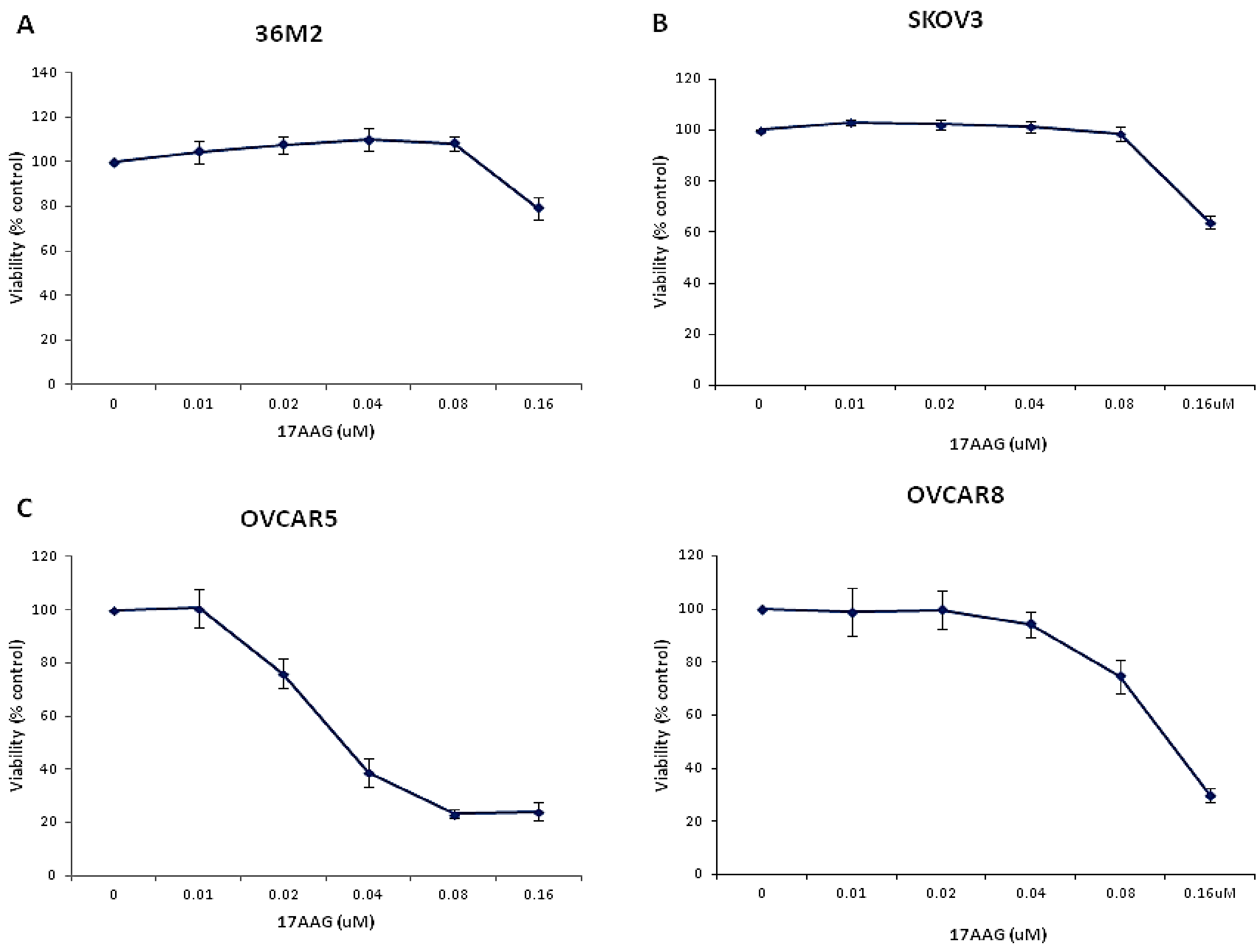

SUPPLEMENTARY FIGURE 1: 17-AAG dose curve in a panel of ovarian cancer cells. (A-D) 17-AAG Dose curves in different ovarian cancer cells. Cells were plated onto a 96-well plate at 1000 cells/well density and treated with indicated concentrations of 17-AAG on the following day. Viability was tested by using CellTiter Glo (Promega) in 5 days. Curves were generated from 3 independent experiments.

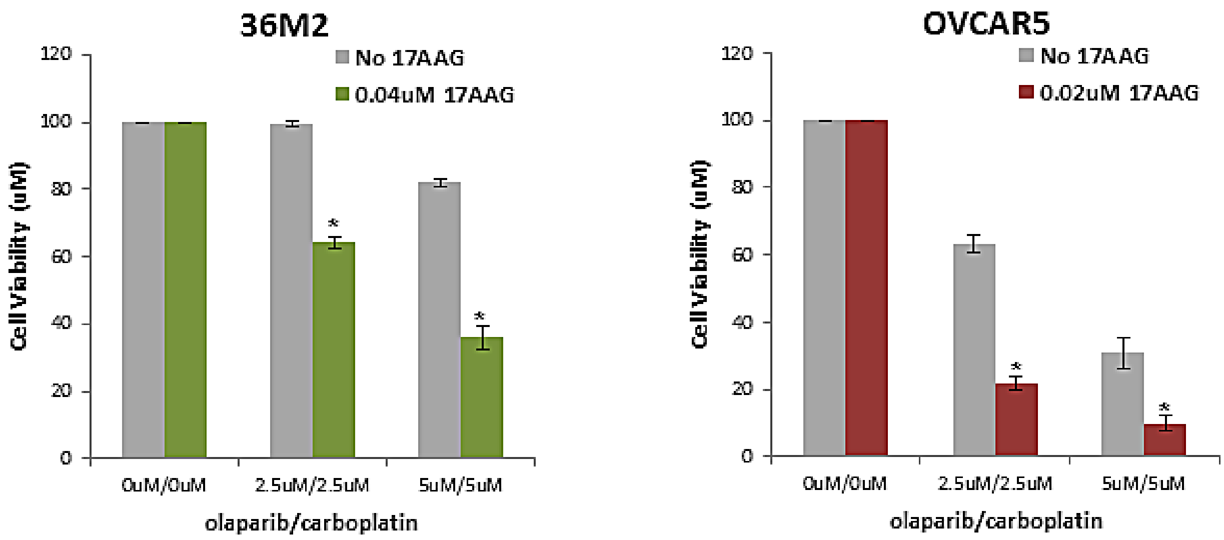

SUPPLEMENTARY FIGURE 2: 17-AAG sensitizes 36M2 and OVCAR5 cells to combination of olaparib and carboplatin. Cells were plated onto a 96-well plate at 1000 cells/well density and treated with indicated concentrations of PARP inhibitor, olaparib and platinum drug, carboplatin on the following day. Viability was tested by using CellTiter Glo (Promega) in 5 days. Plots were generated from 3 independent experiments.
